# Low-dose aspirin in the prevention of preeclampsia in twin pregnancies: A real-world study

**DOI:** 10.3389/fcvm.2022.964541

**Published:** 2023-01-17

**Authors:** Qiongjie Zhou, Xingzhong Zhao, Jinghui Xu, Yu Xiong, Jon F. R. Barrett, Xing-Ming Zhao, Xiaotian Li

**Affiliations:** ^1^Department of Obstetrics, Obstetrics and Gynecology Hospital of Fudan University, Shanghai, China; ^2^Shanghai Key Laboratory of Female Reproductive Endocrine-Related Diseases, Shanghai, China; ^3^Institute of Science and Technology for Brain-Inspired Intelligence, Fudan University, Shanghai, China; ^4^Department of Obstetrics and Gynecology, McMaster University, Hamilton, ON, Canada; ^5^State Key Laboratory of Medical Neurobiology, Institutes of Brain Science, Fudan University, Shanghai, China; ^6^MOE Key Laboratory of Computational Neuroscience and Brain-Inspired Intelligence, and MOE Frontiers Center for Brain Science, Fudan University, Shanghai, China

**Keywords:** twin pregnancy, low dose aspirin, preeclampsia, prevention, China

## Abstract

**Background:**

The use of low-dose aspirin for women with twin pregnancies remains controversial. This study was to describe the frequency of preeclampsia and aspirin use in twin pregnancies in real practice.

**Methods:**

This retrospective cohort study based on real-world data was conducted in the Obstetrics and Gynecology Hospital of Fudan University between 2013 and 2020. Women with twin pregnancies who received prenatal care before 20 weeks of gestational age were included. They were divided into those using low-dose aspirin (LDA group) and those not using aspirin group (N-LDA group). The primary outcome was the frequency of preeclampsia, and secondary outcomes included early-onset and preterm mild and severe preeclampsia.

**Results:**

A total of 2,946 women had twin pregnancies, and 241 were excluded due to missing information. Of 2,705 eligible women, 291 (10.75%) were administered aspirin and the other 2,414 (89.25%) did not. The patients in the LDA group were significantly more likely to be older, have a higher rate of use of ART, have a previous history of hypertension, and have gestational diabetes (*p* < 0.05). In the LDA group, aspirin compliance ≥50% was relatively low (14.43%, 42/291). Preeclampsia occurred in 106 of 291 participants (36.43%) in the LDA group, as compared to 449 of 2,411 (18.62%) in the N-LDA group (OR: 2.15, 95% CI: 1.62–2.82; *p* < 0.01). The association was confirmed (OR: 1.74, 95% CI: 1.26–2.4; *p* < 0.01) in the 1:2 case-matched analysis. Higher odds of ratio in the LDA group were demonstrated (aORs > 1, *p* < 0.01), except for early-onset and preterm mild preeclampsia (*p* > 0.05). This association was confirmed in a subgroup analysis of methods of conception (aORs ≥ 1, *p* > 0.05).

**Conclusion:**

Aspirin prescription of 75 to 100 mg in twin pregnancies was associated with no significant reduction of preeclampsia, which may be due to poor compliance with the aspirin used. Further randomized controlled or prospective cohort studies are required.

## 1. Introduction

Twin gestation is associated with a 2–3 times increased risk of preeclampsia, and low-dose aspirin (LDA) prophylaxis is recommended for its prevention (1–4). Available randomized controlled or cohort studies on the effectiveness of LDA, such as ASPERE, ASPIRIN trial, and the APPEC study, were based on singleton pregnancies, where twins were excluded (5–8). Evidence from a well-designed clinical trial for evaluating the effectiveness of aspirin in the prevention of preeclampsia in twin pregnancies is required.

Studies about the effectiveness of LDA in twin pregnancy are indeterminate. Erkan et al. found that 75 mg aspirin daily did not lower the incidence of preeclampsia or gestational hypertension in twin pregnancy (9); however, 100 mg aspirin daily did reduce the risk in an observational study in China (10). Therefore, whether low-dose aspirin is effective for twin pregnancies in reducing the risk of preeclampsia remains controversial.

Herein, we have conducted a retrospective cohort study in China between 2013 and 2020, for the purpose of describing the frequency of PE and aspirin use in twin pregnancies in a real-world study setting. In this study, the largest of its kind ever conducted, we have compared the incidence of preeclampsia between women with twin pregnancies using and not using LDA. In addition, we have conducted a case-matched analysis and subgroup analysis to further explore the association.

## 2. Methods

### 2.1. Study design and participants

This was a retrospective cohort study of all women with twin pregnancies who received prenatal care before 20 weeks of gestational age in the Obstetrics and Gynecology Hospital of Fudan University in Shanghai, China, from 1 January 2013 to 31 December 2020. Ethical approval was obtained from the research ethics committee of Fudan University (FE21194).

The inclusion criteria for the study were the following: maternal age ≥ 18 years old, twin pregnancies, and first prenatal visit before 20 weeks of gestational age. The exclusion criteria were as follows: first prenatal visit after 20 weeks of gestational age, missing information on the number of fetuses, gestational age, or delivery week.

This cohort of women was divided into two groups: LDA group (women using aspirin) and N-LDA group (women not using aspirin). Indication for LDA was based on the traditional count of clinical risk factors in China (5): women with twin pregnancies are recommended for aspirin administration at 75–100 mg daily dose from 12 to 20 weeks of gestation until 36 weeks of gestation. According to the Chinese guideline, maternal age ≥40 years old, body mass index ≥28 kg/m^2^, use of artificial reproductive technology, and pregnancy interval ≥10 years were considered medium risk factors; and prior history of preeclampsia/fetal growth restriction/placental abruption, renal disease, and hypercoagulable disease were considered high-risk factors. Women with ≥2 medium risk factors or ≥1 high-risk factor were recommended a daily dose of 75–100 mg low-dose aspirin to start between 12 and 20 weeks of gestation.

### 2.2. Variables

Gestational age was confirmed by the measurement of the fetal crown-rump length of the bigger twin in the first-trimester ultrasound scan. Data were extracted from the electronic medical record database. The exposure variable was the use of aspirin. This information was extracted from the electronic medical records about aspirin prescription at the prenatal visit or during hospitalization. Demographic data including maternal age, nulliparity, body mass index (BMI), chorionicity, methods of conception [natural conception and assistant reproductive technology (ART)], previous history of hypertensive disorders, gestational diabetes, delivery week, and delivery mode were recorded. Detailed electronic medical data sources and preprocessing are described in Supplemental Table 1.

### 2.3. Outcome measures

The primary outcome was the frequency of preeclampsia (PE). According to the bulletin of the American College of Obstetricians and Gynecologists' Committee 2019, (3) preeclampsia was diagnosed as the following criteria: (1) Blood pressure: systolic blood pressure >140 mmHg and/or the diastolic blood pressure should be >90 mmHg on at least two occasions 4 h apart developing after 20 weeks of gestation in previously normotensive women; or systolic blood pressure ≥160 mm Hg or diastolic blood pressure ≥110 mm Hg; (2) proteinuria: >300 mg in 24 h or two readings of at least ++ on dipstick analysis of midstream or catheter urine specimens if no 24-h collection is available, or in the absence of proteinuria, new-onset hypertension with the new onset of any of the following: thrombocytopenia [platelet count < 100,000 × 10 (9)/L], renal insufficiency (serum creatinine concentrations > 1.1 mg/dL), impaired liver function (elevated liver transaminases to twice the normal concentration), pulmonary edema, new-onset headache unresponsive to medications, and not accounted for other diagnoses (1, 2).

Secondary outcomes included mild and severe preeclampsia. Severe preeclampsia was defined as any of the following: systolic blood pressure ≥160 mm Hg or diastolic blood pressure ≥110 mm Hg, thrombocytopenia [platelet count < 100,000 × 10 (9)/L], renal insufficiency (serum creatinine concentrations >1.1 mg/dL, or a doubling of the serum creatinine concentration in the absence of other renal diseases), impaired liver function (elevated liver transaminases to twice the normal concentration, or severe persistent right upper quadrant or epigastric pain unresponsive to medication and not accounted for by alternative diagnoses), pulmonary edema, new-onset headache unresponsive to medications, and not accounted for by other diagnoses. Those patients who did not fit the criteria for the diagnosis of severe preeclampsia were classified into mild preeclampsia. Another secondary outcome was the gestational age (GA) at delivery integrated with the occurrence and type of PE as follows: PE delivery at < 34 weeks, PE delivery at < 37 weeks, mild PE delivery at < 34 weeks, mild PE delivery at < 37 weeks, severe PE delivery at < 34 weeks, severe PE delivery at < 37 weeks.

### 2.4. Statement about aspirin

The exposure variable in this study was the administration of low-dose aspirin. The daily low dose was 75–100 mg of aspirin to start between 12 and 36 weeks of gestation. Information about aspirin included whether the participant taking aspirin and adherence as well. Adherence to aspirin was evaluated by the actual prescribed dose/total dose required (from starting gestational week to 36 gestational weeks or delivery week) × 100%, and the daily intake is 75–100 mg.

### 2.5. Statistical analysis

Data are shown as means and standard deviation or numbers (percentages). Differences between the LDA group and N-LDA groups were analyzed using the chi-squared test for categorical variables. A binary logistic regression was employed to evaluate the association, and the results of primary and secondary outcomes were presented as odd ratios (ORs) or as mean differences with 95% of confidence intervals (CIs). The statistically significant variables at the baseline assessment or variables previously reported to be risk factors for preeclampsia were included, such as maternal age, nulliparity, BMI, gestational hypertension, gestational diabetes, gestational week, and ART. In the presence of any significant demographic confounders, adjusted odd ratios (aORs) were calculated after adjusting for the confounders. a *p*-value of < 0.05 was considered statistically significant.

Considering the potential selection bias is likely to lead to a false increased risk of preeclampsia in the LDA group, propensity score matching (PSM) was utilized to reduce the possible bias. PSM was achieved using MatchIt (R package) (11); a conditional logistic regression model was used in 1:2 paired data (after PSM), while an unconditional logistic model was applied in the sensitivity analysis. The variables previously reported to be risk factors for preeclampsia were applied as matching factors, including maternal age, nulliparity, BMI, chorionic type, ART, and previous history of hypertension.

The outcome analysis was calculated using logistic regression, achieved by a generalized linear model (GLM, a R function), with the primary and secondary outcome as the dependent variable and the LDA intake state as the independent variables, and confounding factors that the above factors were matching factors. For the subgroup analysis, we reclassified the data using subgroup metrics (methods of conception) and then examined the association between LDA intake and preeclampsia in a similar way for each subcategory. Missing values in the data were filled with the mean and the plural, respectively, according to the proportion and type of missing values, and details are in the Supplementary material.

### 2.6. Role of the funding source

The funders played no role in the design and conduct of the study; collection, management, analysis, and interpretation of the data; preparation, review, or approval of the manuscript; or decision to submit the manuscript for publication.

## 3. Results

### 3.1. Participants

**Figure 1:**
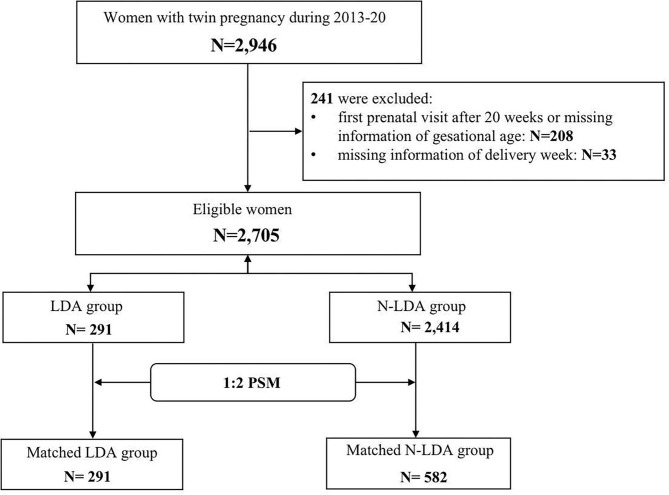
A flow diagram of the selection of participants.

A total of 2,946 women had twin pregnancies and 241 were excluded due to missing information on gestational age or delivery week (Supplemental Table 2). Among 2,705 eligible women, 291 (10.75%) took aspirin (LDA) and the other 2,414 (89.25%) N-LDA did not take aspirin (Figure 1). The patients in the LDA group were significantly more likely to be older, have a higher rate of use of ART, have a previous history of hypertension, and have gestational diabetes (*p* < 0.05). There were no significant differences in parity, BMI, delivery week, and delivery mode between the LDA group and the N-LDA group (Table 1).

**Table 1:** Demographic characteristics of the participants.

	**LDA group (*N* = 291)**	**N-LDA group (*N* = 2,414)**	***P*-value**
Maternal age, yr	32.94 ± 4.34	30.88 ± 4.35	< 0.01
Primiparity, *N* (%)	255 (87.63)	2,038 (84.42)	0.18
BMI, kg/m^2^	27.11 ± 3.82	27.00 ± 3.82	0.75
Chorionicity, *N* (%)		0.51	
Dichorionicity	208 (71.48)	1,669 (69.14)	
Monochorionicity	49 (16.84)	475 (19.68)	
NA	34 (11.68)	270 (11.18)	
Methods of conception, *N* (%)			< 0.01
ART	162 (55.67)	680 (28.17)	
Natural	129 (44.33)	1,734 (71.83)	
Previous history of hypertension, *N* (%)	8 (2.75)	11 (0.46)	< 0.01
Gestational diabetes, *N* (%)	77 (26.46)	482 (19.97)	0.01
Delivery week, weeks	35.57 ± 1.92	35.58 ± 1.92	0.95
Delivery mode, *N* (%)			0.12
Cesarean section	281 (96.56)	2,269 (93.99)	
Vaginal delivery	9 (3.09)	131 (4.50)	
NA	1 (0.34)	14 (4.81)	
Preeclampsia, *N* (%)	101 (34.70)	439 (18.19)	< 0.01
Mild PE, *N* (%)	50 (17.18)	212 (8.78)	< 0.01
Severe PE, *N* (%)	51 (17.53)	227 (9.40)	0.03
PE delivery at < 34 wk, *N* (%)	16 (5.50)	41 (1.70)	< 0.01
PE delivery at < 37 wk, *N* (%)	72 (24.74)	301 (12.47)	< 0.01
Mild PE delivery at < 34 wk, *N* (%)	8 (2.75)	13 (0.54)	< 0.01
Mild PE delivery at < 37 wk, *N* (%)	34 (11.68)	121 (5.02)	< 0.01
Severe PE delivery at < 34 wk, *N* (%)	8 (2.75)	28 (1.16)	0.27
Severe PE delivery at < 37 wk, *N* (%)	38 (13.06)	180 (7.46)	0.02

ART referred to the use of assistant reproductive technology.

In the matched group, BMI, chorionicity, and previous history of hypertension were of no difference between the LDA group and the matched N-LDA group (*p* > 0.05). The rate of ART was observed significantly higher (*p* = 0.03) and maternal age was slightly elder (*p* = 0.03) in the LDA group compared to that in the N-LDA group (Supplemental Table 3). Additionally, aspirin compliance was not satisfactory in the LDA group, since only 14.43% (42/291) of women had compliance of ≥50%, while 85.57% of other women had compliance of < 50%.

### 3.2. Primary outcomes

Preeclampsia occurred in 106 of 291 participants (36.43%) in the LDA group, as compared to 449 of 2,411 (18.62%) in the N-LDA group (OR: 2.15, 95% CI for adjusted OR: 1.62 to 2.82; *p* < 0.01) (Table 2). In the 1:2 case-matched analysis, the association was confirmed (OR: 1.74, 95% CI for adjusted OR: 1.26 to 2.40; *p* < 0.01).

**Table 2:** Outcomes of total participants and case-matched cases.

	**Total participants**						**Case-matched analysis**					
	**LDA group**	**N-LDA group**	**Crude OR (95%CI)**	***P***	**Adjusted OR (95%CI)** ^*^	***P***	**LDA group**	**N-LDA group**	**Crude OR (95%CI)**	***P***	**Adjusted OR (95%CI)**	**P**
**Total participants**												
PE	101 (34.7)	439 (18.2)	2.39 (1.83–3.10)	< 0.01	2.15 (1.62–2.82)	< 0.01	101 (34.7)	137 (23.5)	1.73 (1.27–2.35)	< 0.01	1.74 (1.26–2.40)	< 0.01
Mild PE	50 (17.2)	212 (8.8)	2.15 (1.56–3.06)	< 0.01	1.87 (1.27–2.62)	0.01	51 (17.5)	72 (12.8)	1.47 (1.02–2.20)	0.04	1.48 (0.97–2.12)	0.02
Severe PE	51 (17.5)	227 (9.4)	2.05 (1.21–2.48)	< 0.01	1.88 (1.32–2.64)	< 0.01	50 (17.2)	65 (11.2)	1.69 (1.33–2.51)	< 0.01	1.66 (1.01–2.50)	0.01
**Subtype of PE**												
PE delivery at < 34 wk	16 (5.5)	41 (1.7)	3.37 (1.81–5.97)	< 0.01	3.80 (1.40–6.30)	< 0.01	16 (5.5)	20 (3.4)	1.63 (0.82–3.20)	0.15	2.83 (1.24–6.35)	0.01
PE delivery at < 37 wk	72 (24.7)	301 (12.5)	2.31 (1.71–3.08)	< 0.01	2.17 (1.30–2.53)	< 0.01	72 (24.7)	91 (15.6)	1.77 (1.25–2.51)	< 0.01	1.89 (1.27–2.73)	< 0.01
Mild PE delivery at < 34 wk	8 (2.8)	13 (0.5)	5.22 (2.05–12.5)	< 0.01	6.04 (2.06–17.2)	0.06	8 (2.8)	5 (0.9)	3.26 (1.08–10.90)	0.04	6.07 (1.70–25.70)	< 0.01
Mild PE delivery at < 37 wk	34 (11.7)	121 (5.0)	2.51 (1.66–3.71)	< 0.01	2.38 (1.54–3.59)	< 0.01	34 (11.7)	38 (6.5)	1.89 (1.20–3.18)	< 0.01	1.98 (1.20–3.20)	< 0.01
Severe PE delivery at < 34 wk	8 (2.8)	28 (1.2)	2.41 (1.02–5.10)	0.03	2.26 (0.85–5.48)	0.08	8 (2.8)	15 (2.6)	1.07 (0.43–2.50)	0.88	1.42 (0.51–3.82)	0.48
Severe PE delivery at < 37 wk	38 (13.1)	180 (7.5)	1.86 (1.27–2.68)	0.01	1.69 (1.13–2.48)	< 0.01	38 (13.1)	53 (9.1)	1.50 (0.96–2.33)	0.07	1.54 (0.96–2.44)	0.07
**Subgroup analysis of conception method**												
ART												
PE	66/162 (40.7)	183/680 (26.9)	1.87 (1.30–2.66)	< 0.01	1.73 (1.18–2.52)	< 0.01	66/162 (40.7)	79/278 (28.4)	1.73 (1.15–2.61)	< 0.01	1.59 (1.05–2.43)	0.03
Mild PE	32/162 (19.8)	90/680 (13.2)	1.61 (1.02–2.50)	0.03	1.43 (0.88–2.27)	0.14	32/162 (19.8)	38/278 (13.7)	1.55 (0.93–2.60)	0.09	1.51 (0.88–2.60)	0.13
Severe PE	34/162 (21.0)	93/80 (13.7)	1.08 (1.07–2.58)	0.02	1.62 (1.01–2.54)	0.04	34/162 (21.0)	41/278 (14.8)	1.54 (0.93–2.54)	0.09	1.49 (0.88–2.51)	0.14
**Natural conception**												
PE	35/129 (27.1)	256/1,734 (14.8)	2.15 (1.41–3.21)	< 0.01	2.03 (1.31–3.09)	< 0.01	35/129 (27.1)	58/304 (19.1)	1.58 (0.97–2.55)	0.04	1.60 (1.20–2.60)	0.03
Mild PE	18/129 (14.0)	122/1,734 (7.0)	2.14 (1.22–3.56)	< 0.01	1.99 (1.20–3.40)	0.02	18/129 (14.0)	34/304 (11.2)	1.29 (0.69–2.35)	0.41	1.38 (0.72–2.60)	0.31
Severe PE	17/129 (13.2)	134/1,734 (7.7)	1.81 (1.02–3.03)	0.03	1.72 (0.96–2.91)	0.06	17/129 (13.2)	24/304 (7.9)	1.77 (0.90–3.40)	0.08	1.81 (0.90–3.56)	0.09

Data were present as *N* (%).

* adjusted for maternal age, nulliparity, BMI, gestational hypertension, gestational diabetes, gestational week, and ART.

ART referred to the use of assistant reproductive technology.

### 3.3. Secondary outcomes

The association for secondary outcomes, quantified as the odds ratio in the LDA group with a 95% CI, is shown in Table 2. Higher odds of ratios in the LDA group were demonstrated, including early-onset preeclampsia (delivery at < 34 gestational weeks) and preterm preeclampsia (delivery at < 37 gestational weeks) (aORs > 1, *p* < 0.01), except that there was no significant difference in the odds of ratios for early-onset severe preeclampsia (*p* = 0.48) (Table 2).

### 3.4. Subgroup analysis

A subgroup analysis of methods of conception was conducted to further determine the effect of low-dose aspirin on preeclampsia prevention in twin pregnancy (Table 2). There was a higher risk of preeclampsia in the LDA group regardless of whether ART was used (aORs > 1, *p* < 0.05), together with no difference for mild or severe preeclampsia. In the case-matched analysis, higher odds of ratios were observed in the LDA group for preeclampsia of assistant conception (aORs > 1, *p* < 0.05) and mild preeclampsia of natural conception (OR: 1.99, 95% CI for adjusted OR: 1.20–3.40; *p* = 0.02).

## 4. Discussion

### 4.1. Main findings

In this retrospective cohort study, aspirin prescription of 75–100 mg in twin pregnancies was associated with no significant reduction of preeclampsia, including severe or mild preeclampsia. It may be due to poor compliance and/or insufficient dose of aspirin used for twin pregnancy. Thus, further randomized controlled or prospective cohort studies are required.

### 4.2. Strengths

The main strength of this study is that it provides a real-world setting for describing the frequency of LDA and preeclampsia in twin pregnancy in a real-world setting and further verification by a 1:2 case-matched analysis and a subgroup analysis, which allowed the reduction of the bias as much as possible. Second, previous studies were limited by significant reporting bias (9, 12–14). In this study, adherence to aspirin was extracted from the medical records with quality control, which is expected to be more objective than that originating from self-reporting or questionnaires. Third, varieties of potential confounders, such as maternal age, BMI, parity, and previous history of hypertension, were extracted from the electronic medical database and were well-adjusted.

### 4.3. Limitations

This study had some limitations. First, considering that the patients in the LDA group had more risk factors for preeclampsia than the N-LDA group such as older maternal age, higher rate of use of ART, previous history of hypertension and diabetes, and whether the use of aspirin reduces the risk of preeclampsia in twin pregnancy cannot be inferred. A causal relationship could not infer from our retrospective cohort design, and further randomized controlled design is needed. Second, the aspirin compliance was not so satisfactory that 14.43% of women had compliance of ≥50% in the LDA group, and therefore, this insufficient dose of aspirin used for twin pregnancy potentially contributed to the ineffectiveness of aspirin administration for twin pregnancy in reducing the risk of preeclampsia. Whether a sufficient dose of aspirin is effective needs further assessment. Third, regardless of whether patients who initiated aspirin initiated it before 16 weeks of pregnancy is of importance (15–17), however, the initiation gestational week of aspirin was unavailable in our study. Instead, details about whether aspirin was prescribed and the total prescription dosage were extracted from the medical records for the analysis. Additionally, details regarding potential confounders such as placental growth factor (PlGF) and soluble FMS-like tyrosine kinase-1 (sFlt-1) were not available. Further studies are required to take biological markers such as PlGF and sFlt-1 into consideration.

### 4.4. Interpretation

Our findings indicated that LDA seemed to not lower the risk of preeclampsia in twin pregnancy. Many studies supported that LDA should be used for women at high risk of preeclampsia. Most available evidence of aspirin prophylaxis originated from singleton pregnancies, and those women with multiple pregnancies or those pregnant as a result of ART were excluded. Qualified clinical trials of twin pregnancies covering both natural conception and ART are lacking. This study provided evidence for the fact that low-dose aspirin did not reduce the risk of preeclampsia for twin pregnancies. A possible plausible explanation could be increased fetoplacental demand in twin pregnancies instead of reduced uteroplacental blood supply (18, 19). It is because that increased syncytiotrophoblast stress from one larger placenta or two placentas is more likely to be accounted for preeclampsia in twin pregnancies, instead of maternal underlying cardiovascular phenotype (20). Further studies should be focused on the differential pathological mechanisms.

Another possible reason could be that women with twin pregnancies may require better compliance and a larger dosage to achieve effective prevention of preeclampsia. The different dosage of aspirin is always one of the key concerns. Our study provided evidence for the fact that low-dose aspirin did not reduce the risk of preeclampsia for twin pregnancies. One of the earliest RCTs of aspirin in twin pregnancy, dated 1989, showed that birth weight was increased among 15 women using aspirin compared to 12 who were not using (21). In the 1993 Italian study of 1,106 twin pregnancies, a greater proportion of hypertensive diseases during pregnancy and intrauterine growth restriction was observed in the group of 50 mg/day aspirin, but the difference was insignificant (8). The difference between 75 vs. 100 mg of aspirin, as well as initiation before and after 16 gestational weeks, was not compared since our local guideline recommends a dosage of 75–100 mg before 20 weeks. Aspirin has been demonstrated to be beneficial when given before 16 weeks of gestation and has preventive effect with a 100–150 mg dosage (1–4), and therefore, whether a cutoff of 16 weeks or a larger dose would achieve effective prevention for twin pregnancy requires further validation.

## 5. Conclusion

In conclusion, our findings indicated that an aspirin prescription of 75–100 mg in twin pregnancies was associated with no significant reduction of preeclampsia. This may be due to poor compliance and/or an insufficient dose of aspirin used for twin pregnancy. Further well-designed randomized controlled or prospective cohort studies are required.

## Data availability statement

The raw data supporting the conclusions of this article will be made available by the authors, without undue reservation.

## Ethics statement

The studies involving human participants were reviewed and approved by the Research Ethics Committee of Fudan University, and written informed consent was obtained from all participants (FE21194). The patients/participants provided their written informed consent to participate in this study.

## Author contributions

QZ and XL have full access to all of the data in the study and take responsibility for the integrity of the data and the accuracy of the data analysis, study concept and design, administrative, technical, or material support, and study supervision. QZ, XZ, and XL: drafting of the manuscript and obtained funding. QZ, XZ, JB, and XL: critical revision of the manuscript for important intellectual content. QZ, XZ, X-MZ, and XL: statistical analysis. All authors: acquisition, analysis, or interpretation of data. All authors contributed to the article and approved the submitted version.
