# Supplementary material for: Low-dose aspirin in the prevention of preeclampsia in twin pregnancies: A real-world study
Source: Front Cardiovasc Med. 2023 Jan 17;9:964541. doi: 10.3389/fcvm.2022.964541 (PMC9886671; doi:10.3389/fcvm.2022.964541)
Supplement: Supplementary file 1 [file Data_Sheet_1.PDF]

Low-dose aspirin in prevention of preeclampsia in twin pregnancies: a retrospective cohort study in mainland China

Qiongjie ZHOU, PhD[1.2*]; Xingzhong ZHAO, MS[3*]; Jinghui XU, MS[1], Yu XIONG, PhD[1.2], Jon F R Barrett[4#]; Xing-Ming ZHAO, PhD[3,5, 6#]; Xiaotian LI, PhD[1,2#]

1. Obstetrics and Gynecology Hospital of Fudan University, Shanghai, China

2. Shanghai Key Laboratory of Female Reproductive Endocrine-Related Diseases, Shanghai, China

3. Institute of Science and Technology for Brain-Inspired Intelligence, Fudan University, Shanghai 200433, China

4. Department of Obstetrics and Gynecology, McMaster University, Hamilton, Ontario, Canada

5. Key Laboratory of Computational Neuroscience and Brain-Inspired Intelligence, Fudan University, Ministry of Education, Shanghai 200433, China

6. Fudan ISTBI—ZJNU Algorithm Centre for Brain-inspired Intelligence, Zhejiang Normal University, Jinhua, China.

Supplemental Table 1 Electronic medial data source and preprocessing.

Data items	Data source	Data preprocessing
Aspirin	Extracted from drug prescription information from outpatient and inpatient record.	"1"=use of aspirin from inpatient and (or) outpatient records; "0"=no records of use.
Maternal age	Extracted from diagnosis of inpatient record.	Data were divided into "1"=maternal age≥35, and "0"=maternal age <35. Missing data was completed by mode.
Maternal height	Extracted from diagnosis of inpatient record and checked with obstetrics outpatient registration.	Data were recorded as centimeter. Missing data was completed by mean.
Maternal weight	Extracted from diagnosis of inpatient record and checked with obstetrics outpatient registration.	Data were recorded kilogram. Missing data was completed by mean.
Gestation number	Extracted from diagnosis of inpatient record	Data were recorded as follow: "1"=1408, "2"=684, "3"=295, "4"=141, "5"=56, "6"=11, "7"=9, "8"=2. Missing data was completed by mode.
Parity	Extracted from diagnosis of inpatient record.	Data were divided into "1"=multipara, and "0"=primipara. Missing data was completed by mode
Methods of conception	Extracted from diagnosis of inpatient record to check if assistant reproductive technology (ART) is applied.	Data were divided into "1"=Assistant, and "0"=Natural.
Chorionicity	Extracted from diagnosis of inpatient record.	Data were divided into "1"=monochorionity, "2"=dichorionity.
Preeclampsia	Extracted from diagnosis as "preeclampsia" in inpatient record.	Data were divided into "1"=diagnosed as preeclampsia, and "0"=not diagnosed as preeclampsia.
Severe preeclampsia	Extracted from diagnosis as "severe preeclampsia" in inpatient record.	Data were divided into "1"=diagnosed as severe preeclampsia, and "0"=not diagnosed as severe preeclampsia.
Mile preeclampsia	Extracted from diagnosis as "mild preeclampsia" in inpatient record.	Data were divided into "1"=diagnosed as mild preeclampsia, and "0"=not diagnosed as mild preeclampsia.
Gestational diabetes	Extracted from diagnosis as "gestational diabetes" or "pregestational diabetes" or "pregnancy complicated with diabetes" in inpatient record.	Data were divided into "1"=yes, and "0"=no.
Delivery week	Delivery week is calculated based on birth date and expected date of confinement in electronic medical records.	Delivery week was recorded and analyzed.
Delivery mode	Delivery mode was extracted from diagnosis at delivery.	Data were divided into "1"=Cesarean section, "2"=vaginal delivery.

Supplemental Table 2 Demographic characteristics of included and excluded participants.

		Included (N=2,705)	Excluded (N=241)	P value
Maternal age, yr		31.10±4.16	33±5.32	0.04
Primipara, N(%)		2293(88.69)	205(85.06)	0.10
Delivery week, weeks		35.58±2.13	34.33±1.83	0.59
Delivery mode, N(%)				0.06
	Cesarean section	2550(94.27)	1(0.41)	
	Vaginal delivery	140(5.18)	0(0.00)	
	NA	15(0.55)	240(99.59)	

Data were present as N (%).

Supplemental Table 3 Demographic characteristics of case-matched participants*.

		LDA group (N=291)	Matched N-LDA group (N=582)	P value
Maternal age, yr		32.94±4.35	32.29±4.35	0.03
Primiparity, N(%)		255(87.63)	497(85.40)	0.43
BMI, kg/m^2		27.11±3.82	27.26±3.82	0.64
Chorionicity, N(%)				0.63
	Dichorionicity	208(71.48)	398(68.38)	
	Monochorionicity	49(16.84)	106(18.21)	
	NA	34(11.68)	78(13.40)	
Methods of conception, N(%)				0.03
	ART	162(55.67)	278(47.77)	
	Natural conception	129(44.33)	304(52.23)	
Previous history of hypertension, N(%)		8(2.75)	8(1.37)	0.25
Gestational diabetes, N(%)		77(26.46)	141(24.23)	0.52
Delivery week, weeks		35.57±1.92	35.51±1.92	0.64
Delivery mode, N(%)				0.12
	Cesarean section	281(96.56)	541(92.96)	
	Vaginal delivery	9(3.09)	33(11.34)	
	NA	1(0.34)	8(2.75)	
Preeclampsia, n (%)		101(34.7)	137(23.54)	<0.01
Mild PE, N(%)		51(17.53)	72(12.37)	0.05
Severe PE, N(%)		50(17.18)	65(11.17)	0.13
PE delivery at <34wk, N(%)		16(5.5)	20(3.44)	0.21
PE delivery at <37wk, N(%)		72(24.74)	91(15.64)	<0.01
Mild PE delivery at <34wk, N(%)		8(2.75)	5(0.86)	0.06
Mild PE delivery at <37wk, N(%)		34(11.68)	38(6.54)	<0.01
Severe PE delivery at <34wk, N(%)		8(2.75)	15(2.58)	0.05
Severe PE delivery at <37wk, N(%)		38(13.06)	53(9.1)	0.31

ART: assistant reproductive technology.

*case-matched by maternal age, maternal BMI, chorionicity, pregnancy complications, delivery week and delivery mode.
